# Long-term skeletal and dental effects of facemask versus chincup treatment in Class III patients: A retrospective study

**DOI:** 10.1007/s00056-017-0083-3

**Published:** 2017-03-24

**Authors:** B. Wendl, M. Stampfl, A. P. Muchitsch, H. Droschl, H. Winsauer, A. Walter, M. Wendl, T. Wendl

**Affiliations:** 1 Department of Oral Surgery and Orthodontics, Medical University Graz, Graz, Austria; 2 Private Practice, Bregenz, Austria; 3 Departamento de Ortodoncia y Ortopedia dento-facial, Universitat Internacional de Catalunya, Barcelona, Spain; 4 Institute of Software Development and Biomedical Engineering, Technical University Graz, Graz, Austria

**Keywords:** Class III syndrome, Facemask, Chincup, Orthopedic appliances, Longterm stability, Klasse III Syndrom, Gesichtsmaske, Kopf-Kinn-Kappe, Kieferorthopädische Apparaturen, Langzeitstabilität

## Abstract

**Objectives:**

To investigate the skeletal and dental changes during chincup versus facemask treatment, to compare the long-term effects of the two appliances, and to document the impact of each on treatment success.

**Methods:**

In all, 61 patients with Class III syndrome were retrospectively analyzed at three examination times: 7.8 ± 1.7 years of age (T0, pretreatment), 9.6 ± 2.4 years of age (T1, posttreatment), and around 15–20 years later (T2, long-term follow-up).

**Results:**

Significant changes of specific cephalometric parameters for all treatment times: T0–T1 (SNA, interbase and gonial angle, Björk’s sum angle, maxillomandibular differential, and distance of upper lip to esthetic line), T1–T2 (NL-NSL, SNB, mandibular-body length, effective mandibular length, and effective maxillary length), and T0–T2 (mandibular-body length, effective mandibular length, effective maxillary length, maxillomandibular differential, SNB, ANB, gonial angle, Björk’s sum angle, and Wits appraisal). The T1–T2 results illustrate that in both treatment groups the typical Class III growth pattern often reappeared after treatment, including gains in SNB angle, condylion-gnathion length, and gonion-menton distance.

**Conclusions:**

Either a facemask or a chincup may be effectively used to treat Class III malocclusion. There were differences in long-term stability. Maxillary development was similarly favorable in both groups of patients with successful outcome. The subgroup in whom chincup treatment had failed were mainly characterized by excessive mandibular growth, or lack of maxillary catch-up growth, with deterioration of the maxillomandibular relationship notably in the initial phase of treatment. Early chincup treatment did not have an adverse impact on the temporomandibular joints.

## Zusammenfassung

**Ziele:**

Es sollten die skelettalen und dentalen Veränderungen bei Therapie mit Kopf-Kinn-Kappe beziehungsweise Gesichtsmaske untersucht, die Langzeitbehandlungseffekte beider Apparaturen verglichen, und der Einfluss der jeweiligen Therapiegeräte auf den Therapieerfolg dokumentiert werden.

**Methoden:**

Insgesamt wurden Daten von 61 Patienten mit Klasse-III-Syndrom zu 3 Untersuchungszeitpunkten retrospektiv analysiert: im Alter von 7,8 ± 1,7 (T0, vor der Behandlung) und 9,6 ± 2,4 Lebensjahren (T1, nach der Behandlung) sowie etwa 15–20 Jahre später (T2, langfristiges Follow-up).

**Ergebnisse:**

Signifikante Veränderungen spezifischer kephalometrischer Parameter zeigten sich für alle Therapieintervalle: T0–T1 (SNA, Interbasis- und Gonion-Winkel, Winkelsumme nach Björk, maxillomandibuläre Differenz und Distanz Oberlippe-Ästhetiklinie), T1–T2 (NL-NSL, SNB, Länge des Unterkiefers, effektive Mandibula- und effektive Maxillalänge) sowie T0–T2 (Länge des Unterkiefers, effektive Mandibula- und effektive Maxillalänge, maxillomandibuläre Differenz, SNB-, ANB-, Gonionwinkel Winkelsumme nach Björk und Wits-Appraisal). Die T1–T2-Ergebnisse beider Behandlungsgruppen zeigen, dass das typische Klasse III Wachstumsmuster nach Therapie häufig wieder auftritt. Dabei zu beobachten sind u. a. eine Zunahme des SNB-Winkels, der Kondylion-Gnathion Länge sowie der Gonion-Menton Distanz.

**Schlussfolgerungen:**

Sowohl die Gesichts-Maske, als auch die Kopf-Kinn-Kappe sind effektive Geräte zur Behandlung eines Klasse III Syndroms. Unterschiede bestehen bezüglich der langfristigen Stabilität der Behandlungsergebnisse. Ähnlich der Gesichtsmaskentherapie zeigte sich interessanterweise, auch bei erfolgreicher Kopf-Kinn-Kappen-Behandlung, eine günstige Oberkieferentwicklung. Die Subgruppe, bei der die Kopf-Kinn-Kappen Behandlung ohne Erfolg blieb, zeichnete sich im Wesentlichen durch zu starkes Unterkieferwachstum beziehungsweise einer Verschlechterung der maxillo-mandibulären Differenz bei fehlendem catch-up-Wachstum des Oberkiefers, vor allem in der initialen Therapiephase, aus. Eine Frühbehandlung mit der Kopf-Kinn-Kappe hatte keinen ungünstigen Einfluss auf die Kiefergelenke.

## Introduction

Early treatment of Class III malocclusion is provided with different protocols reflecting the specific nature of the syndrome, which may consist in maxillary retrognathism, mandibular prognathism, or a combination of both [34]. Available options range from intraoral removable appliances such as inclined planes or maxillary protrusive plates, through functional orthopedic appliances like Fränkel’s function regulator III or the Class III bionator (also known as “reversed bionator”), to extraoral appliances like a facemask or a chincup [5]. In addition, the older the Class III patients, the more likely their treatment will involve modalities of skeletal anchorage [37].

The function regulator III was proposed by Fränkel for patients with maxillary retrognathism [10, 15]. A facemask, by contrast, both includes a protrusive force vector acting upon the maxillary complex and exerts a retrusive force on the mandible, thus, being used in Class III patients with growth problems in both jaws [4, 8, 9, 16, 17, 25]. A chincup [2, 11, 31] is used primarily in patients who exhibit moderate prognathism of the mandible (SNB: >80°; condylion-gnathion: >120 mm in girls and >134 mm in boys) and a maxilla of normal dimensions in a correct anteroposterior position [7, 21]. In addition to its favorable effects in the sagittal and vertical planes, a chincup also influences the craniobasal structures, thus, potentially modifying the position of the glenoid fossa [1, 19, 24, 27].

Key factors to the success of skeletal Class III treatment also include the degree of the anomaly and the hereditary pattern [35]. The present study was designed to analyze changes of skeletal and dental cephalometric parameters during chincup or facemask treatment, to compare the long-term stability of these changes, and to determine whether both appliances may affect the success of outcome in different ways.

## Materials and methods

A total of 61 patients, all of whom had been diagnosed with Class III syndrome prior to treatment, were retrospectively evaluated (Table 1). Two examiners independently analyzed data from cephalograms, casts, and orthopantomograms reflecting each patient’s pretreatment situation at a mean age of 7.8 ± 1.7 (range 5–9) years (T0), posttreatment situation after correction of the malocclusion at 9.6 ± 2.4 (range 9–11) years (T1), and long-term follow-up situation 15–20 years later (T2). The cephalometric tracings were based on landmarks from various analysis schemes (Björk, Jarabak, Jacobsen, McNamara) and included 21 (2 dental, 17 skeletal, 2 soft-tissue) parameters.

**Tab. 1:** Class III patients classified by treatment appliances and treatment success **Tab. 1** Klasse-III-Patienten, eingeteilt nach Behandlungsapparaturen und Therapieerfolg

Facemask (success)		Chincup (success)		Chincup (failure)	
*n* = 23		*n* = 25		*n* = 13	
Male: *n* = 15	Female: *n* = 8	Male: *n* = 15	Female: *n* = 10	Male: *n* = 11	Female: *n* = 2

The chincup group was compiled from the patient data on file at the Department of Orthodontics at Medical University Graz, where a chincup is the preferred method of treating Class III cases. The data for the facemask group were made available from an external database in a private practice. For each patient, complete pretreatment (T0), posttreatment (T1), and long-term (T2) records were available, the latter comprising follow-up periods of up to 20 years. Only patients were included who, at T0, exhibited skeletal and dental signs of Class III syndrome (negative overjet, Wits <−1 mm, negative ANB difference, Class III malocclusion). Cleft lip and palate or any other syndromes led to exclusion. Chincups were worn at a force of 600 g per side for 24 h a day whenever possible and, once a positive overjet was achieved, overnight. The facemask patients wore an additional expansion appliance (with an acrylic splint from the upper canines to the first molars). In the correction stage, the mask was worn throughout the day with a force of approximately 300 g applied. The elastics were inserted at an angle of approximately 60° to the occlusal plane.

Treatment success was defined as positive overjet and overbite (≥ 1 mm) and no transverse crossbite. As no facemask treatment failures were available from the external database, only the patient subgroup with failed chincup treatment (chincup_failure_) was compared to the subgroup with successful treatment (chincup_success_). Follow-up data of untreated Class III patients or Class I patients from the literature [22] were used for statistical comparisons of deviations from normal values and were compared in accordance with the age change. In the chincup group, the patients’ temporomandibular joints were assessed at each time (T0, T1, and T2) by two independent examiners clinically using functional analysis and visually on the panoramic radiographs using criteria by Hatcher [12]. IBM SPSS Statistics Version 22” (2013) was used for descriptive statistical analysis of data. Differences were considered significant at *p* ≤ 0.05. A *t* test for connected samples was calculated to compare mean values, and one-way analysis of variance was applied for intergroup comparisons.

## Results

Significant dental and skeletal changes occurred within the various treatment groups, and the soft-tissue parameters were also found to change considerably. Interestingly, both mandibular and maxillary growth was more pronounced after treatment in the successful chincup group than in the facemask group. Table 2 lists the descriptive statistics of the linear and angular cephalometric parameters measured, subdivided into the three treatment groups (facemask_success_, chincup_success_, chincup_failure_) and broken down by examination times T0, T1, and T2. Table 3 lists the results of the intergroup comparisons with the differences measured over each of the three intervals between the three examination times (T0–T1, T0–T2, T1–T2).

**Tab. 2:** Descriptive statistics (Mean and SD) of cephalometric skeletal and dental parameters measured in the facemask_success_ versus the chincup (chincup_success_/chincup_failure_) groups at different examination times, including pretreatment (*T0*), posttreatment (*T1*), and 15–20-years follow-up (*T2*) **Tab. 2** Deskriptive Statistik (Mittewerte und SD) der kephalometrischen skelettalen und dentalen Parameter, die in der Gruppe Gesichtsmaske_Erfolg_ und den Gruppen Kinnkappe (Kinnkappe_Erfolg_/Kinnkappe_kein Erfolg_) zu den Untersuchungszeitpunkten - vor Therapie (*T0*), nach Therapie (*T1*) und 15–20 Jahre nach  Therapie (*T2*) vermessen wurden

Parameter	Time	Facemask		Chincup				Parameter	Time	Facemask		Chincup			
		Success		Success		Failure				Success		Success		Failure	
		Mean	SD	Mean	SD	Mean	SD			Mean	SD	Mean	SD	Mean	SD
SNA (°)	T0 T1 T2	79.1 80.5 79.6	4.1 4.3 4.0	78.2 78.0 79.6	3.0 3.0 4.6	78.2 78.9 80.5	4.1 3.7 6.3	Cond.-A (mm)	T0 T1 T2	81.7 85.0 89.4	4.6 5.4 5.8	77.4 80.7 89.8	5.2 5.9 5.9	77.7 80.9 86.8	6.5 6.2 7.5
SNB (°)	T0 T1 T2	78.7 78.0 79.0	3.8 3.9 3.6	78.3 77.6 80.1	2.6 2.7 4.0	79.5 78.9 83.2	4.5 3.7 5.6	Cond.-Gn (mm)	T0 T1 T2	111.2 114.4 126.0	6.7 7.6 11.6	102.9 107.2 125.6	7.2 9.1 9.7	103.7 110.0 124.1	9.0 11.9 10.6
ANB (°)	T0 T1 T2	0.4 2.1 0.8	3.4 2.7 2.2	−0.1 0.4 −0.3	1.6 1.8 2.1	−1.3 0.0 −2.7	3.3 3.5 2.9	MM diff. (mm)	T0 T1 T2	29.6 29.5 36.8	4.7 4.9 7.3	25.5 26.5 35.7	4.4 5.6 6.1	26.0 29.0 37.7	5.7 8.0 5.2
Ar-Go-Me (°)	T0 T1 T2	130.5 129.8 129.2	6.1 5.4 6.0	129.4 125.3 121.1	5.0 5.8 7.3	131.5 130.3 127.9	3.1 4.5 6.6	Ar-Go (mm)	T0 T1 T2	41.8 42.6 50.2	3.8 4.1 5.8	38.8 40.5 50.2	5.6 5.9 7.6	38.5 40.9 51.7	2.9 4.9 6.6
NSBa (°)	T0 T1 T2	128.1 127.6 128.4	4.7 6.2 6.6	127.3 127.1 126.7	7.1 6.8 6.7	126.5 126.6 125.5	5.6 6.2 5.6	Go-Me (mm)	T0 T1 T2	67.6 70.8 77.2	8.3 5.4 8.1	63.4 67.8 79.1	6.0 6.0 6.7	63.6 67.4 75.3	8.5 8.2 8.5
ML-NSL (°)	T0 T1 T2	37.3 38.6 36.7	5.7 5.9 5.4	33.8 33.7 30.7	4.1 5.1 6.5	34.6 33.9 30.1	4.7 4.2 6.1	Spp-Spa (mm)	T0 T1 T2	51.8 52.2 56.7	3.6 2.7 8.4	49.0 52.1 55.5	3.6 3.9 4.5	49.6 51.2 54.3	3.5 3.0 3.5
NL-NSL (°)	T0 T1 T2	8.6 7.4 9.1	3.2 5.0 4.6	7.5 8.1 7.3	3.5 2.8 4.7	8.5 7.5 6.3	3.5 4.2 3.8	UCI/SN (°)	T0 T1 T2	100.8 104.4 106.5	9.0 7.1 5.0	99.8 102.5 109.9	10.4 9.2 6.6	100.6 107.0 111.4	9.9 10.4 9.3
ML-NL (°)	T0 T1 T2	28.9 33.1 29.8	6.4 10.7 11.3	26.9 25.6 23.5	5.0 4.9 5.9	26.5 26.5 24.0	5.3 4.9 5.9	LCI/ML (°)	T0 T1 T2	83.8 83.7 85.7	7.0 6.4 5.9	85.1 86.5 90.1	6.9 6.9 5.3	85.5 82.3 89.4	8.8 10.4 13.9
Björk’s sum (°)	T0 T1 T2	397.0 398.4 396.7	5.5 6.0 4.8	394.1 392.7 389.4	4.5 5.3 7.0	394.9 393.5 389.8	4.9 4.7 5.9	UL-EL (mm)	T0 T1 T2	−4.5 −2.9 −5.4	2.6 3.0 3.4	−3.6 −3.8 −6.2	2.4 3.1 3.4	−5.2 −4.6 −7.9	4.4 3.2 3.4
Wits (mm)	T0 T1 T2	−5.4 −2.2 −2.6	2.9 2.0 2.2	−3.9 −1.9 −2.5	2.2 2.3 2.5	−3.5 −1.9 −4.6	3.3 3.3 2.2	LL-EL (mm)	T0 T1 T2	−1.7 −1.1 −2.7	2.6 3.0 3.5	−1.1 −1.8 −3.6	2.8 2.9 2.8	−0.8 −1.4 −2.1	3.4 2.6 3.4
PFH:AFH (ratio)	T0 T1 T2	61.8 60.9 62.8	4.9 4.8 4.2	62.6 63.6 67.4	4.4 4.9 5.8	62.0 63.4 67.3	4.8 3.6 5.4								

*EL* esthetic line, *LCI* lower central incisor, *LL* lower lip, *UCI* upper central incisor, *UL* upper lip, *PFH:AFH* posterior facial height:anterior facial height

**Tab. 3:** Statistically significant differences of cephalometric skeletal and dental parameters measured in the facemask_success_ versus the chincup (chincup_success_/chincup_failure_) groups during treatment (*T0*–*T1*), during the long-term follow-up of 15–20 years (*T1*–*T2*), and throughout the observation period (*T0*–*T2*) **Tab. 3** Statistisch signifikante Unterschiede der skelettalen und dentalen Fernröntgen -Messwertdifferenzen, die in der Gruppe Gesichtsmaske_Erfolg_ und den Gruppen Kinnkappe (Kinnkappe_Erfolg_/Kinnkappe_kein Erfolg_) im Behandlungszeitraum (*T0*–*T1*), während des 15–20 Jahre Follow-up nach Behandlungsabschluss (*T1*–*T2*) und im Zeitraum (*T0*–*T2*) ermittelt wurden

	T0–T1						T0–T2						T1–T2					
	Facemask		Chincup				Facemask		Chincup				Facemask		Chincup			
	Success		Success		Failure		Success		Success		Failure		Success		Success		Failure	
SNA (°)	+1.4	*	−0.3	*	+0.7	n.s.	+0.8	n.s.	+1.5	n.s.	+2.3	n.s.	−0.6	*	+1.7	*	+1.5	*
SNB (°)	−0.7	n.s.	−0.7	n.s.	−0.5	n.s.	+0.4	*	+2.0	*	+3.7	*	+1.3	*	+2.7	*	+4.3	*
ANB (°)	+1.7	n.s.	+0.5	n.s.	+1.3	n.s.	+0.7	*	−0.4	*	−1.5	*	−1.3	n.s.	−0.8	n.s.	−2.7	n.s.
Ar-Go-Me (°)	−0.7	*	−4.1	*	−1.3	n.s.	−1.4	*	−8.3	*	−3.6	n.s.	−0.7	n.s.	−3.9	n.s.	−2.4	n.s.
NSBa (°)	−0.5	n.s.	−0.2	n.s.	+0.1	n.s.	−0.1	n.s.	−1.2	n.s.	−1.1	n.s.	+0.5	n.s.	−1.1	n.s.	−1.2	n.s.
ML-NSL (°)	+1.3	n.s.	−0.1	n.s.	−0.7	n.s.	−0.6	n.s.	−3.1	n.s.	−4.5	n.s.	−2.3	n.s.	−2.8	n.s.	−3.8	n.s.
NL-NSL (°)	−1.2	n.s.	+0.7	n.s.	−0.9	n.s.	+0.6	n.s.	−0.2	n.s.	−2.2	n.s.	+1.9	*	−0.5	*	−1.3	*
ML-NL (°)	+4.2	*	−1.3	*	0.0	n.s.	+0.8	n.s.	−3.3	n.s.	−2.5	n.s.	−4.1	n.s.	−2.2	n.s.	−2.5	n.s.
Björk’s sum (°)	+1.4	*	−1.4	*	−1.5	n.s.	−0.5	*	−4.8	*	−5.1	*	−2.1	n.s.	−3.2	n.s.	−3.7	n.s.
Wits (mm)	+3.2	n.s.	+2.1	n.s.	+1.5	n.s.	+2.9	*	+1.3	n.s.	−1.2	*	−0.6	n.s.	−0.9	n.s.	−2.7	n.s.
PFH:AFH ratio	−0.9	n.s.	+1.1	n.s.	+1.4	n.s.	+0.9	n.s.	+4.9	n.s.	+5.3	n.s.	+2.3	n.s.	+3.7	n.s.	+3.9	n.s.
Cond-A (mm)	+3.2	n.s.	+3.3	n.s.	+3.2	n.s.	+8.3	*	+12.5	*	+9.1	n.s.	+4.9	*	+9.1	*	+5.9	*
Cond-Gn (mm)	+3.3	n.s.	+4.3	n.s.	+6.3	n.s.	+15.2	*	+22.8	*	+20.4	n.s.	+12.0	*	+18.7	*	+14.1	*
MM diff. (mm)	−0.2	*	+1.0	n.s.	+3.0	*	+6.9	*	+10.3	n.s.	+11.7	*	+7.2	n.s.	+9.6	n.s.	+8.7	n.s.
Ar-Go (mm)	+0.7	n.s.	+1.7	n.s.	+2.5	n.s.	+8.3	n.s.	+11.2	n.s.	+13.3	n.s.	+7.7	n.s.	+9.6	n.s.	+10.8	n.s.
Go-Me (mm)	+3.3	n.s.	+4.4	n.s.	+3.7	n.s.	+10.2	*	+15.9	*	+11.6	n.s.	+6.6	*	+11.5	*	+7.9	*
Spp-Spa (mm)	+0.5	n.s.	+3.1	n.s.	+1.5	n.s.	+5.3	n.s.	+6.8	n.s.	+4.6	n.s.	+4.5	n.s.	+3.7	n.s.	+3.1	n.s.
UCI/SN (°)	+3.6	n.s.	+2.8	n.s.	+6.4	n.s.	+6.1	n.s.	+10.0	n.s.	+10.7	n.s	+2.7	n.s.	+7.9	n.s.	+4.4	n.s.
LCI/ML (°)	−0.1	n.s.	+1.4	n.s.	−3.2	n.s.	+2.6	n.s.	+4.3	n.s.	+3.9	n.s.	+2.6	n.s.	+2.9	n.s.	+7.1	n.s.
UL-EL (mm)	+1.6	*	−0.2	*	+0.5	n.s.	−0.7	n.s.	−2.8	n.s.	−2.7	n.s.	−2.4	n.s.	−2.7	n.s.	−3.3	n.s.
LL-EL (mm)	+0.6	n.s.	−0.7	n.s.	−0.5	n.s.	−0.9	n.s.	−2.7	n.s.	−1.3	n.s.	−1.6	n.s.	−2.2	n.s.	−0.7	n.s.

*EL* esthetic line, *LCI* lower central incisor, *LL* lower lip, *UCI* upper central incisor, *UL* upper lip, *PFH:AFH* posterior facial height:anterior facial height

* Differences were considered significant at p ≤ 0.05

## Cephalometric developments during treatment and through the observation period

The SNA angle changed most significantly (by +1.4°) in the facemask group during T0–T1, then decreasing back by 0.9° while increasing by 1.6° in the chincup group during T1–2. SNB angle decreased by about 0.7° with both appliances during T0–T1 but increased more markedly (by 2.5°) in the chincup than in the facemask group (1°) during T1–T2; in the chincup_failure_ group, this angle increased by >4°. ANB angle improved by 1.7° in the facemask group during T0–T1, thus, approaching the ideal range; during T1–T2, however, the jaw relationship again deteriorated. Gonial angle decreased by 4.1° in the chincup group during T0–T1, then decreasing further for a total change of 8.3° throughout T0–T2. NSBa decreased slightly in the facemask and in the chincup group during T0–T1, followed by continuation of the downward trend in the chincup group versus an increase back to almost normal in the facemask group during T1–T2. Interbase angle (ML-NL) decreased by 1.3° in the chincup group—thus, counteracting the vertical growth tendency—while increasing by 4.2° in the facemask group during T0–T1. Björk’s sum angle decreased, corresponding to the extreme gonial-angle decrease, in the chincup group but increased in the facemask group during T0–T1; during T1–T2, the values decreased in both groups and more markedly so in the chincup group.

Wits appraisal increased by 3.2 mm, such that an almost neutral jaw relationship was reached, in the facemask group compared to 2.1 mm in the chincup group during T0–T1; both groups showed similar decreases during T1–T2. Effective maxillary length (Cond-A) increased in both groups by the same amounts (3.2 or 3.3 mm) during T0–T1, followed by further gains of 4.4 mm in the facemask and 9.1 mm in the chincup group during T1–T2. Effective mandibular length (Cond-Gn) was found to increase less in the facemask group than in the chincup group throughout T0–T2. Maxillomandibular differential decreased slightly in the facemask group during T0–T1 and increased by about 3 mm more in the chincup than in the facemask group throughout T0–T2. Mandibular-body length (Go-Me) showed larger increase in the chincup group than in the facemask group throughout T0–T2. Upper-incisor inclination (UCI/SN) was characterized by more pronounced camouflage positions in the chincup group at T2, whereas lower-incisor inclination was almost normal by that time. Distance of upper lip to esthetic line (UL-EL) decreased by 1.6 mm in the facemask group during T0–T1.

Visual examination of the panoramic radiographs did not reveal any remarkable findings at the various examination times (T0, T1, and T2). Three patients showed condylar changes, including, in one case, identification of a flattened condyle at T2, which, however, had been present previously and did not deteriorate during treatment; one suspicious dorsal formation of a condyle; one flattening of the right condyle. None of the patients revealed any clinical signs or symptoms meeting the criteria of a functional anomaly as defined by the Graz dysfunction index.

## Discussion

Evidence has repeatedly been provided that a start of treatment as early as possible is essential to the success of Class III treatment [2, 3, 29, 36]. Other authors have suggested a low efficiency of Class III appliances [20, 21]. Due to the natural growth direction of the nasomaxillary complex, treatment with a facemask should be expected to yield the most pronounced skeletal effects up to 8 years of age [9, 13]. In older patients, the dentoalveolar effect will progressively increase [14]. Additional use of a maxillary expansion appliance is known to boost the skeletal efficiency of a facemask [6], and this approach was also used in the facemask group of the present study.

While the treatment effects of a facemask are well documented [2–4, 16, 18, 19, 25, 29, 38], long-term data are scarce. Most studies have reported increases in ANB angle, overjet, Cond-A, and SNA angle a decrease in maxillomandibular differential, an improvement of the molar relationship, and clockwise rotation of the mandible [18, 22, 32, 38]. Shanker et al. [30] did not observe a significant difference in A-point changes during Class III therapy of Chinese children with a facemask and an expansion appliance compared to an untreated control group. We found an A-point change of +1.4° in our facemask group during treatment (T0–T1). The ANB angle improved by 1.7° in our facemask group, and Wits appraisal, too, revealed an almost neutral jaw relationship at T1, yet the intermaxillary relationship again deteriorated over the further course of growth (T1–T2). Ngan et al. [21] studied changes in a Chinese Class III population treated with a facemask and an expansion appliance. They identified slight movement of the maxilla but no significant movement of the mandible in the sagittal or vertical plane. Our study revealed SNB reductions by 0.7° with both appliances (facemask and chincup) from T0 to T1.

Mitani and Fukazawa [20] investigated the effect of chincup treatment on 26 Japanese girls. They found that complete inhibition of mandibular growth was difficult to achieve and the treatment effects to vary greatly between individuals. Regardless of the duration of daily force application and of age categories, they noted an increase in mandibular length. Our study, too, revealed increases in mandibular-body length and effective length of the mandible—in both treatment groups, albeit more so in the chincup than in the facemask group.

Sugawara et al. [31] studied the long-term effects of chincup treatment in three different age groups. They noted profile improvements in the early treatment stage but, since many of these improvements failed to remain stable, did not recommend treatment with a chincup alone for skeletal Class III patients exhibiting an additional maxillary growth deficit in the sagittal plane. Yoshida et al. [39] studied the combined use of a maxillary protractor and a chincup in 28 Japanese girls. They found significant increases in SNA by 2.6° with advancement and counterclockwise rotation of the maxilla, as compared to decreases in SNB by 1.31° with clockwise rotation and delayed growth of the mandible, followed by a relapse of about 35% with the mandible showing excessive growth while its improved position was maintained.

Wendell et al. [33] arrived at clearly successful outcomes of chincup treatment. They analyzed 10 children of an intermediate age (about 8.1 years) treated for a mean of 3.1 years and compared the results to untreated Class I and Class III subjects both after treatment and 6.2 years later. Overall, they found the mandibular growth rate to be 60–68% lower than in untreated control groups. In the literature, the effect and stability of mandibular growth inhibition by chincup treatment has been controversially discussed [28, 29, 33]. Outcomes seem to be more stable in girls than in boys with Class III [23]. It has been suggested that the compression force exerted by a chincup corrects the direction of jaw growth by influencing the mitotic activity of the prechondroblast zone in the temporomandibular joints [35]. Also, Class III treatment might affect growth by modifying condylar morphology and the glenoid fossa [19, 26]. Our study does not support documented findings of a vertical ramus-length reduction [11, 27].

Despite these findings, the efficiency of chincup treatment is not uncontroversial, especially with regard to the risk of causing harm to the condyles. The long-term follow-up and clinical examinations in our study demonstrated no indications of craniomandibular dysfunction in any of the patients. Deguchi and McNamara [8] did not observe any changes of the temporomandibular joints, either. Uçüncü et al. [32], in a retrospective study of cases with combined maxillary retro- and mandibular prognathism, compared the treatment effects of a chincup (12 patients aged 11.03 years) versus a Delaire mask (12 patients aged 10.72 years). They found improvements in ANB angle molar relationship, and overjet in both groups, as well as significantly greater improvements of the sagittal position of the maxilla and of the molar relationship in the group treated by maxillary protraction. Our study revealed changes of the maxilla in both treatment groups, which even were more pronounced in the chincup_success_ than in the facemask group.

## Conclusions

Early treatment of Class III syndrome led to successful outcomes both with chincup and with facemask appliance. Successful chincup treatment has similarly favorable effects on maxillary development as treatment with a facemask. The initially successful outcomes do, however, differ with regard to their long-term stability. Failed outcomes of chincup treatment are mainly due to uncontainable growth of the mandible with deterioration of the maxillomandibular differential. Early chincup treatment was not observed to have an adverse impact on the temporomandibular joints.
